# Abdominal Wall Abscess after Prophylactic Percutaneous Endoscopic Gastrostomy Placement in a Patient Undergoing Chemoradiotherapy for Laryngeal Cancer

**DOI:** 10.1002/deo2.70259

**Published:** 2026-02-16

**Authors:** Ryuji Okamoto, Hironori Sunakawa, Yuji Owaki, Hiroaki Oka, Yuta Hoshi, Susumu Okano, Tomonori Yano

**Affiliations:** ^1^ Department of Gastroenterology and Endoscopy National Cancer Center Hospital East Kashiwa‐shi Japan; ^2^ Department of Head and Neck Medical Oncology National Cancer Center Hospital East Kashiwa‐shi Japan; ^3^ Division of Science and Technology for Endoscopy Exploratory Oncology Research and Clinical Trial Center, National Cancer Center Kashiwa‐shi Japan

**Keywords:** abdominal wall abscesses, chemoradiotherapy, complication, head and neck cancer, percutaneous endoscopic gastrostomy

## Abstract

Prophylactic percutaneous endoscopic gastrostomy (PEG) placement for nutritional management prior to chemoradiotherapy is a common procedure in patients with head and neck cancer, for which serious complications are rare. Herein, we present a case of abdominal wall abscess that developed 12 days following prophylactic PEG placement in a patient with laryngeal cancer. This rare complication was initially difficult to diagnose due to subtle abdominal symptoms. In this case, antibiotic treatment alone was inadequate, necessitating drainage tube insertion. This case highlights that delayed diagnosis can lead to necrotizing fasciitis, a potentially fatal condition that should be considered in the differential diagnosis.

## 1 Introduction

Percutaneous endoscopic gastrostomy (PEG) is a well‐established procedure with a high success rate; however, mild complications occur in 18%–38% of cases and major complications in 2%–4% [1]. Although abdominal wall abscess is a potential complication of PEG placement, it is relatively rare. The immunosuppressive effects of chemoradiotherapy (CRT) increase the risk of PEG‐related infections, making such complications clinically significant despite their rarity [2]. This report presents a case of an abdominal wall abscess that occurred after PEG tube placement using the introducer technique, which is a widely used and relatively safe method.

## 2 Case Report

A man in his 50s presented with persistent hoarseness. Analysis of a biopsy obtained from the right vocal cord and radiological imaging, the patient was diagnosed with laryngeal squamous cell carcinoma (staged as cT3N0M0 according to the UICC classification, eighth edition). Definitive CRT was planned in accordance with the patient's wish for laryngeal preservation, and a prophylactic PEG was placed for nutritional support. His medical history included angina pectoris treated with catheterization, dyslipidemia, sudden‐onset left‐sided sensorineural hearing loss, a smoking history of 15 cigarettes per day until 1 year prior to carcinoma diagnosis, and cessation of heavy alcohol use in his 40s.

PEG was performed using the MIC‐KEY Introducer Kit (AVANOS Medical Inc., Alpharetta, GA, USA), and a balloon‐button gastrostomy tube (MIC‐KEY 18 Fr, 4.0 cm; AVANOS) was placed after three points of gastropexy. Cefazolin was administered preoperatively for infection prophylaxis and continued for 3 days postoperatively. The procedure took approximately 20 min with minimal blood loss.

**FIGURE 1:**
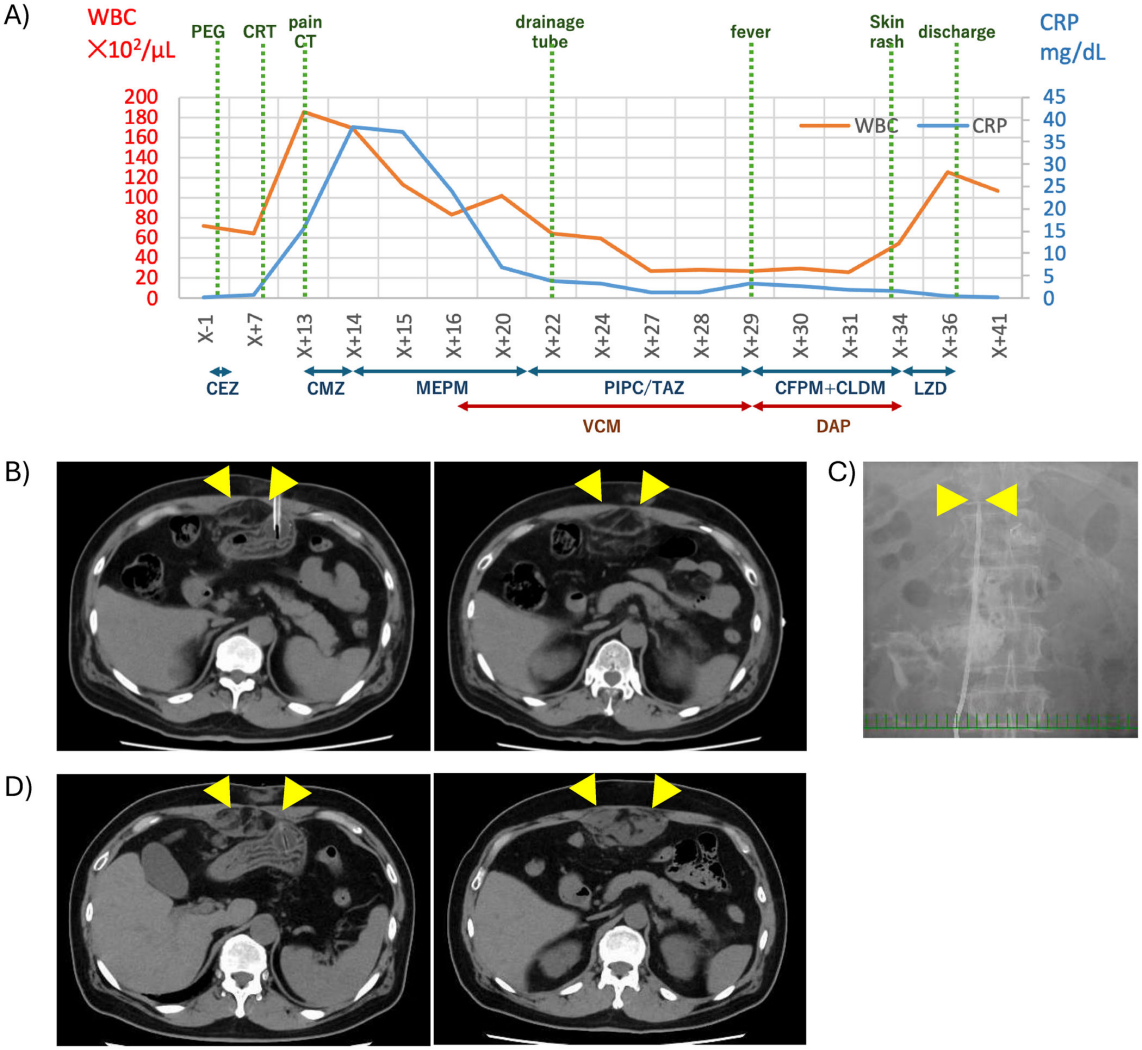
Changes in WBC and CRP levels and CT imaging findings. (A) Trends in WBC and CRP over time. PEG placement was performed on day X. CRT was started on X+8, but the patient developed abdominal pain on X+12, with worsening symptoms and increases in WBC and CRP; CT on X+13 revealed an abdominal wall abscess, and cefmetazole (CMZ) was initiated. Symptoms worsened on X+14, prompting a change to meropenem (MEPM), with subsequent improvement in WBC and CRP. Antibiotics were switched to piperacillin/tazobactam (PIPC/TAZ) on X+21. Follow‐up CT on X+22 showed enlargement of the abscess, and a drainage tube was placed. On X+29, drug‐induced fever was suspected, and antibiotics were changed to cefepime (CFPM), clindamycin (CLDM), and daptomycin (DAP). Thereafter, WBC and CRP decreased, and the patient was discharged on X+36. (B, D) CT scan images on postoperative days (POD) 12 and 22. The arrowhead indicates the abscess cavity, which had enlarged on POD 22 compared with POD 12. (C) Abdominal X‐ray showing the placement of the drainage tube. The arrowhead indicates the drainage tube tip. CEZ, cefazolin; CFPM, cefepime; CLDM, clindamycin; CMZ, cefmetazole; CRP, C‐reactive protein; CRT, chemoradiotherapy; CT, computed tomography; DAP, daptomycin; LZD, linezolid; MEPM, meropenem; PEG, percutaneous endoscopic gastrostomy; PIPC/TAZ, piperacillin‐tazobactam; VCM, vancomycin; WBC, white blood cell.

The patient experienced mild pain at the gastrostomy site for the first 3 days without signs of bleeding or infection. The postoperative course was uneventful, and oral intake was initiated on postoperative day (POD) 3. As planned, CRT was initiated on POD 8. Although the pain at the PEG insertion site had improved, the patient developed upper abdominal pain on POD 12. On POD 13, purulent exudate was observed around the gastrostomy site. The patient reported worsening abdominal pain, and laboratory tests indicated elevated inflammatory markers: white blood cell (WBC) count of 18,500/µL and a C‐reactive protein level of 15 mg/dL (Figure 1A). Computed tomography (CT) revealed soft tissue shadows around the gastrostomy site and peritoneal thickening, which led to a diagnosis of an abdominal wall abscess (Figure 1B). The patient's vital signs were stable, with only mild abdominal and cutaneous findings. Computed tomography showed no evidence of gas formation, and necrotizing fasciitis was therefore considered unlikely.

Cefmetazole was initiated, and the patient was placed on fasting. Blood cultures were obtained, and exudate was collected from the PEG insertion site for culturing. The following day, the patient was switched to meropenem because of worsening pain and temporary loss of consciousness. On POD 15, laboratory results showed a decrease in the WBC count. On POD 16, the exudate cultures showed *Staphylococcus aureus* and *Pseudomonas aeruginosa*. Vancomycin was added to cover gram‐positive organisms. On POD 21, meropenem was changed to piperacillin‐tazobactam based on the results of antimicrobial susceptibility testing. Follow‐up CT on POD 22 revealed an enlarged soft tissue density in the abdominal wall. The radiology department was consulted, and ultrasound‐guided drainage was performed (Figure 1C,D). On POD 23, upper GI endoscopy confirmed no communication between the stomach and abscess cavity, after which oral intake was resumed.

On POD 26, the patient developed a high fever (> 38°C). Suspecting a drug‐induced fever, the antibiotics were changed to cefepime, clindamycin, and daptomycin on POD 29. The fever resolved the following day, and the patient was discharged on POD 36 after completing treatment without removal of the PEG tube; antibiotics were switched to linezolid on POD 34 and stopped at discharge. Following interruption of CRT due to infection, radiotherapy alone was selected to minimize the risk of recurrent infection associated with myelosuppression, as well as to accommodate the patient's wish for laryngeal preservation. The infection was well‐controlled after discharge. Radiotherapy was discontinued at the patient's request, but no residual or recurrent disease was detected.

## 3 Discussion

Abdominal wall abscesses typically occur secondary to malignancies, inflammatory diseases, or surgical site infections. These abscesses are often difficult to diagnose and manage [3]. In many cases, antibiotic treatment alone is insufficient, and surgical drainage is necessary. Shiihara et al. [3] described a complicated case of a primary abdominal wall abscess, and Payne‐James et al. [4] reported a delayed diagnosis owing to the absence of fever in a post‐PEG abscess.

The incidence of PEG site infections reportedly ranges from 4% to 30%, while known risk factors include diabetes, malignancy, and immunosuppressive therapy. Patients with head and neck cancers are at particularly high risk [5]. CRT, which impairs immune function, is also recognized as a significant contributor to infection risk [6, 7]. Chemotherapy may lead to neutropenia and bone marrow suppression, resulting in immunosuppression. It can also damage anatomical barriers such as the skin and mucosa, making patients more susceptible to infection. Since PEG placement is another risk factor for infection and abscess formation, the combination of these factors was thought to have contributed to abscess development in this patient [8].

Inadequate adhesion between the gastric serosa and abdominal wall is one major cause of abdominal wall abscess following PEG placement, which can lead to serious complications, including contamination and necrotizing fasciitis [9, 10]. In the report by Ditesheim et al., patients who developed abdominal wall abscesses typically presented with symptoms within one week after PEG placement, with most cases occurring within four days [9]. In the present case, although the initial postoperative course was uneventful, infection developed shortly after CRT initiation, indicating that CRT may have increased the patient's susceptibility to infection and contributed to abscess formation.

In the present case, an abdominal wall abscess developed following the initiation of chemotherapy after PEG placement. However, it resolved with conservative, multidisciplinary management after diagnosis based on clinical findings, which enabled subsequent radiotherapy. Although rare, abdominal wall abscess is a potentially serious PEG‐related complication, particularly in cancer patients treated with systemic chemotherapy. Careful assessment, including ultrasound or CT imaging, should be employed when clinically suspected.

## Author Contributions

**Ryuji Okamoto**: data curation, writing – original draft, and writing – review and editing. **Hironori Sunakawa**: supervision, project administration, writing – original draft, and writing–review and editing. **Yuji Owaki**: review and editing. **Hiroaki Oka**: review and editing. **Yuta Hoshi**: writing – review and editing. **Susumu Okano**: supervision and writing – review and editing. **Tomonori Yano**: supervision and writing – review and editing.

## Funding

The authors received no specific funding for this work.

## Conflicts of Interest

The authors declare no conflicts of interest.

## Ethics Statement

All procedures were conducted in accordance with the ethical principles outlined in the Declaration of Helsinki and its subsequent revisions.

## Consent

Patient consent was obtained on an opt‐out basis.
